# Safety, tolerability, and pharmacokinetics of TG-1000, a new molecular entity against influenza virus: first-in-human study

**DOI:** 10.3389/fphar.2023.1272466

**Published:** 2023-11-01

**Authors:** Su-Mei Xu, Li-Wen Chang, Cheng-Yuan Tsai, Wan-Li Liu, Dai Li, Shan-Shan Li, Xiao-Min Li, Ping-Sheng Xu

**Affiliations:** ^1^ Phase I Clinical Trial Center, Xiangya Hospital, Central South University, Changsha, China; ^2^ National Clinical Research Center for Geriatric Disorders, Xiangya Hospital, Central South University, Changsha, China; ^3^ TaiGen Biotechnology Co., Ltd., Taipei, China

**Keywords:** first-in-human, TG-1000, influenza, safety, tolerability, pharmacokinetics

## Abstract

**Background:** The cap-snatching mechanism of influenza virus mRNA transcription is strongly suppressed by TG-1000, a prodrug rapidly metabolized into TG-0527, is a potent cap-dependent nucleic acid endonuclease inhibitor. Herein, we aimed to assess the safety, tolerability, and pharmacokinetics of TG-1000 in healthy participants and the effect of food on the pharmacokinetics and safety of TG-1000.

**Method:** The study was divided into 2 parts: Part A [Single Ascending-Dose (SAD) study, 10–160 mg] and Part B [Food-Effect (FE) study, 40 mg] were launched sequentially. The study included 66 participants for both investigations. We administered different TG-1000 capsules or placebo doses per the study protocol and collected blood samples for pharmacokinetic assessments at specific times. In plasma, TG-1000 and its active metabolite TG-0527 were assayed, and PK parameters were determined.

**Results:** In SAD, the increase in AUC was less than the proportional increase in dose over the 20–160 mg dose range, while the increase in C_max_ was proportional to the increase in dose. In the 10–160 mg dose range, T_1/2_, λz and T_max_ of TG-0527 were dose-independent; and T_1/2_ and T_max_ were within 33.8–39.4 h and 3.02–6 h, respectively. In FE, the AUC_0-inf_, AUC_0-last_, and C_max_ of TG-0527 decreased by approximately 17.52%, 18.76%, and 41.35%, respectively, and the T_max_ delay was around 1.50 h. No serious adverse events occurred during the studies.

**Conclusion:** Overall, TG-1000 was well tolerated and exhibited an acceptable safety and PK profile, supporting further clinical investigation of TG-1000 for the treatment of influenza.

## 1 Introduction

Influenza remains one of the world’s greatest public health challenges. Every year across the globe, there are an estimated 1 billion cases, of which 3 to 5 million are severe cases, resulting in 290,000 to 650,000 influenza-related respiratory deaths (WHO, 2019). Even though some adamantanes, neuraminidase inhibitors (NAIs), and cap-dependent endonuclease (CEN) inhibitor have been approved for the treatment of influenza (CDC, 2022); however, all have limitations. Adamantanes (including amantadine and rimantadine) are not recommended for treating influenza because of widespread antiviral resistance (CDC, 2022). NAIs (including oseltamivir, zanamivir, and peramivir) are widely used; however, they are effective only within 48 h of symptom onset (Ison, 2017), may associated with insufficient decreases in virus titers and prolonged virus shedding (Treanor et al., 2000; Lee et al., 2013; Kondo et al., 2016), and emergence with strain mutations in seasonal or avian viruses that led to the development of NAI resistance (Takashita et al., 2016; Zhang et al., 2017). Baloxavir is CEN inhibitor; however, the emergence of viruses with PA/I38X substitutions was associated with transient rises in infectious virus titers, prolongation of virus detectability, initial delay in symptom alleviation, and uncommonly with symptom rebound (Uehara et al., 2019). Thus, there is a clear need for alternative antiviral drugs with potential to overcome resistance issues, not only for the treatment of seasonal influenza but also to have available as a treatment option for future pandemics.

TG-1000, prodrug of TG-0527, is a new molecular entity (NME) and a potent cap-dependent endonuclease inhibitor. Currently, TG-1000 is under development by TaiGen Biotechnology for the treatment of acute uncomplicated influenza in adult patients with potential advantages: 1) Against A, B influenza virus, H7N9 avian influenza virus are effective; 2) Oral dose, both therapeutic and preventive functions; 3) It can inhibit existing neuraminidase-resistant disease strains; 4) Resistant to drug resistance.

The *in-vitro* IC_50_ of TG-0527, active form of TG-1000, is 5.69 nM against cap-snatching mechanism of influenza viral mRNA transcription. It is also active against influenza virus growth, with EC50 values range from 0.35 nM to 3.10 nM against influenza A viruses and from 2.75 nM to 11.90 nM against influenza B viruses. In cytotoxicity assays when tested up to 100 μM, TG-0527 displayed an excellent safety profile. In preclinical *in-vivo* studies, TG-1000 is rapidly converted to an avtive form TG-0527 by hydrolysis. All plasma concentrations of TG-1000 were below the lower limit of quantification (LLOQ, 0.5 ng/mL) at all sampling points. In infection mouse model, and oral administration of 5–25 mg/kg TG-1000 showed a significantly reduction (more than 2 logs) in lung viral titers and demonstrated potent antiviral efficacy in achieving high survival rates.

Therefore, a phase I clinical TG-1000 investigation was conducted in healthy human participants in China to assess its safety, tolerability, and Pharmacokinetic (PK) profile and also evaluated the food effect in this study.

## 2 Methods and materials

### 2.1 Study design

Healthy volunteers were enrolled in this Phase I, single-center, randomized, double-blind, placebo-controlled, single-dose escalation investigation to evaluate the safety, tolerability, and PK profile of TG-1000 as well as to evaluate the pharmacokinetic effects of food on a single oral TG-1000 dose (NCT04495322, ClinicalTrials.gov). The study was divided into two parts: Part A, a randomized, double-blind, placebo-controlled, sequential, single oral dose escalation design; and Part B, a randomized, open, dual-treatment (fasting vs. full stomach), dual-cycle, dual-sequence crossover design. The tests were performed at Xiangya Hospital’s Phase I Clinical Trials Research Center, Central South University. Before the study commenced, approval was obtained from the study’s Independent Ethics Committee (IEC). Additionally, written informed consent was obtained from participants before enrollment into the trials.

### 2.2 Participants

The inclusion criteria were as follows; 1) Healthy male or female adults aged 18–45 years; 2) A strict Body Mass Index (BMI) of between 19.0 and 24.0 kg/m^2^ (including the threshold); and 3) Body weight ≥50 kg and ≥45 kg for men and women, respectively. The exclusion criteria included; 1) Clinically significant cardiac, cerebrovascular, and gastrointestinal diseases; 2) Participants with a history of blood donation or tobacco and alcohol abuse in the 3 months preceding the first TG-1000 dose; 3) Participants who underwent surgery in the previous 6 months; 4) Participants under any medication; 5) Fertility potential in women; and 6) Men unwilling to use effective contraception.

### 2.3 Procedures

Part A (SAD): This study section was planned to comprise 6 dose groups:10 mg, 20 mg, 40 mg, 80 mg, 120 mg, and 160 mg. Group A1 (10 mg) had 4 eligible participants receiving TG-1000 administered as a single 10 mg oral dose to evaluate the initial TG-1000 safety and PK profile. The remaining dose groups (A2 to A6) each had 10 eligible participants randomized in an 8:2 ratio and received TG-1000 treatment or placebo in a single oral dose. All study medications were administered on an empty stomach. The study doses were evaluated sequentially in increments from the lowest dose group (10 mg), progressing group by group to the highest dose group. Before proceeding to the next dose group, a safety evaluation of the preceding dose group and an interim PK assessment had to be completed within 72 and 24 h of dosing, respectively.

Part B study (FE): A 40 mg TG-1000 dose was selected (based on Part A preliminary results) for the food effect study. In this section, there were two treatment sequence groups (treatment sequence AB for group B1 and treatment sequence BA for group B2), each with 6 healthy volunteers (3 males and 3 females). A total of 12 eligible participants were equally randomized to either group B1 or B2 to receive TG-1000. A typical two-sequence, two-cycle (cycle 1 or cycle 2) crossover design in which participants received treatment A first, followed by treatment B (Sequence AB), and treatment B first, followed by treatment A (sequence BA), was employed. Treatment A was a single oral TG-1000 dose in the fasted state (after 10 h of overnight fasting), while Treatment B was a single oral TG-1000 dose in the satiated state (30 min after a high-fat, high-calorie breakfast). Based on preliminary PK data from Part A of the study, the elimination half-life of TG-0527 was approximately 40 h. Consequently, the elution period between the two treatments was set to ≥14 days (approximately eight times the elimination half-life).

### 2.4 Safety assessments

The safety assessments included Adverse Events (AEs), vital signs, physical examination, Electrocardiogram (ECG), and laboratory safety testing. All safety data, including AEs, laboratory safety tests, electrocardiograms, physical examinations, and vital signs, must be listed and summarized with descriptive statistics during safety evaluations. Descriptive statistics were also used to summarize demographic information and baseline characteristics. The safety data were summarized based on the treatment group to which the participants were assigned in the Safety Population (SP). All clinical adverse events, laboratory tests, or other test results were evaluated for severity using the National Cancer Institute (NCI) Common Toxicity Criteria (CTCAE) version 5.0.

### 2.5 Biological sample collection

Approximately 4 mL of blood samples were collected at the scheduled times for analysis. In Part A, peripheral blood serum samples were collected before and at 0.5, 1, 1.5, 2, 2.5, 3, 3.5, 4, 5, 6, 8, 12, 24, 36, 48, 60, 72, 96, 120, and 168 h after dosing. Blood serum samples were collected at 336 h post-dosing for participants receiving the 40 mg, 80 mg, 120 mg, and 160 mg dose groups. On the other hand, during Part B, peripheral blood samples were obtained before and at 0.5, 1, 1.5, 2, 2.5, 3, 3.5, 4, 5, 6, 8, 12, 24, 36, 48, 60, 72, 96, 120, and 168 h post-dosing. Potassium oxalate-sodium fluoride anticoagulant was used in sample collection.

To separate the plasma, the collected blood samples were centrifuged for 10 min at 4°C, 2,700 × g, and subsequently divided into two centrifuge tubes (≥0.7 mL plasma each), frozen, and stored under −80°C awaiting analysis.

### 2.6 Bioanalytical procedures

To determine the concentration of TG-1000 and its active form of TG-0527 in human plasma, a simple, precise, and accurate LC-MS/MS method was developed and validated. The internal standard for TG-1000 was designated as TG-0601402. The regression coefficient was 0.9968 under the 1–300 ng/mL calibration range. The intra-run and the inter-run precisions ranged from 0.4% to 8.3% and 2.1%–5.0%, respectively. The accuracies ranged between 96.7% and 104.2%. The selected internal standard for the metabolite TG-0527 was TG-0601401. The calibration curves were linear over the 2–600 ng/mL range, with a 0.9977 regression coefficient. The intra-run and the inter-run precisions ranged from 1.5% to 5.7% and 1.9%–3.9%, respectively. The accuracies were between 99.2% and 104.3%.

### 2.7 Pharmacokinetic assessments

A Non-Compartmental (NCA) model by WinNonlin Software version 8.3.1 (Pharsight, Cary, NC, United States) was used to calculate PK parameters of TG-0527. The calculated Pharmacokinetic parameters included: Maximum blood concentration (C_max_), Time to reach C_max_ (T_max_), Area Under the Curve from zero time to a specific time (AUC_0-t_), Area Under the Curve from zero time to the last quantifiable concentration (AUC_0-last_), Area Under the Curve from zero time to infinity (AUC_0-inf_), Terminal clearance half-life (T_1/2, z_), Apparent overall clearance (CL/F), Apparent total volumetric distribution (V/F), Cumulative excretion (A_e_), and Fractional excretion (F_e_). A Linear Up Log Down (LULD) method was used to calculate AUC_0-t_ and AUC_0-∞_, whereas T_max_ and C_max_ were based on actual measured values.

### 2.8 Statistical analysis

All statistical analyses were performed using SAS 9.4 statistical software. For continuous variables, descriptive statistics were determined, including the number of cases (missing cases), mean, median, standard deviation, P25 (25th percentile) and P75 (75th percentile), and maximum and minimum values. The number of decimal places for maximum and minimum values matched the original data, whereas the other parameters were rounded to an additional decimal place based on the number of decimal places in the original data. Descriptive statistics for categorical variables included the number of cases and percentage of participants (1 decimal place reserved). If the number of cases was zero, no percentages were calculated. If no specific denominator was available, the total number of participants in the corresponding subgroup of the analyzed population was used to calculate percentages.

Pharmacokinetics parameters (AUC_0-t_, AUC_0-inf_, and C_max_) were evaluated to assess the dose proportionality relationship, while non-dose dependent parameters were assessed by evaluating T_max_, T_1/2,z_, and λ_z_. A linear misfit test was used to assess the linearity between the log-transformed PK parameters and the natural logarithm of the dose. The slope parameter β, 95% Confidence Interval (CI), and a two-sided test (H0:β = 1 or H0:β = 0) were estimated as appropriate. A dose proportionality relationship is shown if β = 1 for the dose-dependent parameters (AUC_0-t_, AUC_0-inf_, and C_max_) and β = 0 for the non-dose-dependent parameters (T_max_, T_1/2,z_, and λ_z_). The dose-dependent parameters were normalized based on the dosage. The statistical model’s assumption of normality and variance chi-square were assessed through residual analysis. The geometric means of AUC_0-inf_, AUC_0-t_, and C_max_ were log-transformed to perform the food impact assessment. Based on the log-transformed data, a 90% CI of the geometric mean ratio of AUC_0-inf_, AUC_0-t,_ and C_max_ at satiation and fasting within the 80%–125% equivalence limit indicated the absence of food effects. The Wilconxon paired test was used to analyze T_max_ differences.

## 3 Results

### 3.1 Demographic profile

Herein, 66 eligible participants (54 in the single ascending-dose study and 12 in the food-effect study) were enrolled and completed the study. No participants were discontinued from the study. Table 1 summarizes the demographic characteristics of all enrolled participants.

**TABLE 1:** Demographics and baseline characteristics for the enrolled participants.

	Single ascending-dose study					Food effect study			
	Placebo (N = 10)	10 mg (N = 4)	20 mg (N = 8)	40 mg (N = 8)	80 mg (N = 8)	120 mg (N = 8)	160 mg (N = 8)	B1* (N = 6)	B2* (N = 6)
age, year	26.1 (5.9)	21.8 (4.3)	20.1 (1.8)	23.8 (7.1)	23.5 (4.0)	21.5 (3.1)	24.9 (6.4)	24.4 (4.9)	26.5 (6.4)
Female/male, n	5/5	2/2	4/5	4/5	5/5	4/4	4/4	4/3	3/3
Height, cm	162.55 (10.27)	165.38 (5.09)	168.38 (8.68)	164.78 (4.68)	163.80 (7.11)	163.56 (6.84)	161.81 (9.36)	164.07 (10.39)	167.17 (5.42)
Weight, kg	56.28 (8.56)	55.50 (4.94)	62.32 (8.16)	58.39 (5.18)	57.54 (5.68)	58.58 (5.55)	55.80 (5.82)	56.63 (5.85)	60.85 (4.82)
BMI, (kg/m^2^)	21.20 (1.50)	20.25 (1.24)	21.91 (1.22)	21.48 (1.49)	21.43 (1.48)	21.89 (1.39)	21.30 (1.21)	21.03 (0.93)	21.75 (0.55)

BMI, body mass index. Note: Data are expressed as mean (SD), except for gender, which is shown as n/n.

^a^ Group B 1, and B2 experienced two treatment periods, respectively. Group B1: fasting→high-fat meal. Group B 2: high-fat meal→fasting.

### 3.2 Pharmacokinetics properties

All plasma concentrations of TG-1000 are below the lower limit of quantification (LLOQ, 1 ng/mL) at all sampling points by validated LC-MS/MS method.

Part A; Single Ascending-Dose (SAD) study: Following oral administration, TG-1000 was rapidly hydrolyzed to its active form of TG-0527. TG-0527 was detected and used to calculate the PK parameters in this study.

**FIGURE 1:**
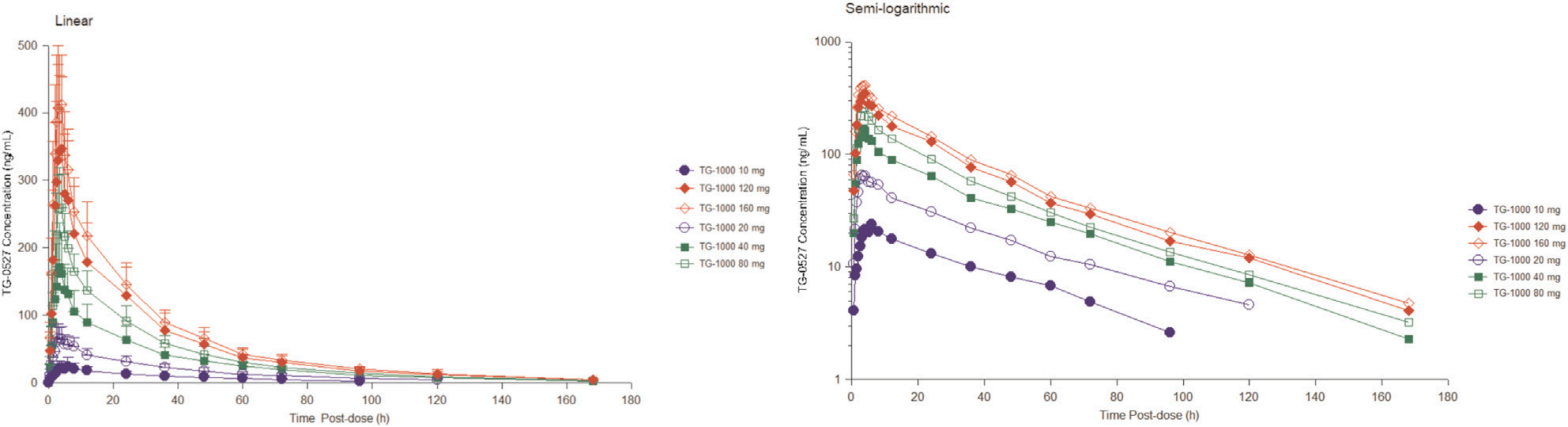
The mean plasma TG-0527 concentration-time curves in each dose group after a single dose of TG-1000 (left: linear coordinates, right: semi-logarithmic coordinates). Mean (SD) of plasma TG-0527 concentration is displayed in this figure.

Table 2 summarizes the PK parameters of TG-0527 in each dose group following administration a single TG-1000 dose. Figure 1 demonstrates the mean plasma TG-0527 concentration-time curves.

**TABLE 2:** The PK parameters of TG-0527 in each dose group after administration of a single dose of TG-1000.

Pharmacokinetic parameters	10 mg (N = 4)	20 mg (N = 8)	40 mg (N = 8)	80 mg (N = 8)	120 mg (N = 8)	160 mg (N = 8)
AUC_0-t_ (h∙ng/mL)	1,180 (18.9)	2,390 (31.4)	4,730 (24.1)	6,580 (20.6)	8,600 (33.4)	10,300 (19.3)
AUC_0-∞_ (h∙ng/mL)	857 (46.3)	2,210 (33.6)	4,540 (25.3)	6,420 (20.4)	8,370 (33.6)	10,100 (19.1)
C_max_ (ng/mL)	22.2 (60.8)	65.2 (36.2)	171 (31.7)	273 (17.0)	348 (34.5)	446 (14.5)
T_max_ (h)*	6.00 (4.00,6.00)	3.50 (2.50,5.00)	3.50 (3.00,5.00)	3.50 (3.00,6.00)	3.27 (2.50,4.00)	3.02 (2.00,4.00)
T_1/2, z_ (h)	38.3 (23.4)	39.4 (12.6)	33.8 (15.6)	34.4 (10.8)	33.8 (7.9)	33.8 (9.0)
CL/F (L/h)	8.49 (18.9)	8.38 (31.4)	8.46 (24.1)	12.2 (20.6)	14.0 (33.4)	15.5 (19.3)
V_z_/F (L)	423 (32.1)	476 (23.3)	413 (31.6)	603 (25.0)	682 (28.8)	757 (15.2)
CL_R_ (mL/h)	26.2 (50.3)	25.9 (31.0)	27.0 (28.0)	30.3 (44.4)	22.1 (45.9)	25.7 (26.6)
Urine						
Ae (mg)	0.0269 (98.5)	0.0621 (31.4)	0.134 (27.5)	0.214 (46.8)	0.204 (54.6)	0.286 (25.0)
Feu_0-t_ (%)	0.269 (98.5)	0.311 (31.4)	0.335 (27.5)	0.268 (46.8)	0.170 (54.6)	0.179 (25.0)

AUC_0-t_ area under the concentration–time curve from time zero to the time of the last measurable concentration, AUC_0-∞_ area under the concentration–time curve from time zero to infinity, C_max_ maximum observed plasma concentration, T_max_ time to maximum plasma concentration, t_1/2_ terminal elimination half-life, V_z_/F apparent distribution volume, CL/F clearance rate, Ae cumulative excretion, Feu fractional excretion. Note: Data are expressed as geometric mean (coefficient of variation, CV%), except for T_max_, which is shown as median (min, max).

The dose linear relationship was assessed using Confidence Interval (CI) criteria. Table 3 depicts the dose proportionality analysis. For the 20–160 mg dose interval, the slope estimate (β) and its 90% CI for C_max_, AUC_0-t_, and AUC_0-∞_ were 0.88 (0.76–1.01), 0.70 (0.59–0.81), and 0.68 (0.57–0.79), respectively. Over the 20–160 mg dose range, the increase in AUC was less than the proportionate increase in dose, but the increase in C_max_ was proportional to the increase in dose. The median of T_max_ and the mean t_1/2_ were within 3.02–6.00 h and 33.8–39.4 h, respectively (Table 2).

**TABLE 3:** The dose proportionality analysis for C_max_ and AUC of plasma TG-0527 in a single ascending-dose study.

PK parameters	Dose interval	Slope estimate (β) (90% CI)	Lack of fit *p*-value	Conclusion
AUC_0-t_ (h∙ng/mL)	10–160 mg	0.80 (0.70,0.91)	0.0313^a^	Inconclusive
AUC_0-∞_ (h∙ng/mL)	10–160 mg	0.73 (0.64,0.82)	0.2121	Less than dose proportionality
C_max_ (ng/mL)	10–160 mg	1.00 (0.88–1.12)	0.0094^a^	Inconclusive
AUC_0-t_ (h∙ng/mL)	20–160 mg	0.70 (0.59–0.81)	0.3463	Less than dose proportionality
AUC_0-∞_ (h∙ng/mL)	20–160 mg	0.68 (0.57–0.79)	0.4069	Less than dose proportionality
C_max_ (ng/mL)	20–160 mg	0.88 (0.76–1.01)	0.0655	Dose Proportionality

^a^ Assumption of linearity violated (lack of fit *p*-value ≤ 0.05) CI, confidence interval Model: ln (parameter) = intercept + slope × ln (dose) + random error.

**FIGURE 2:**
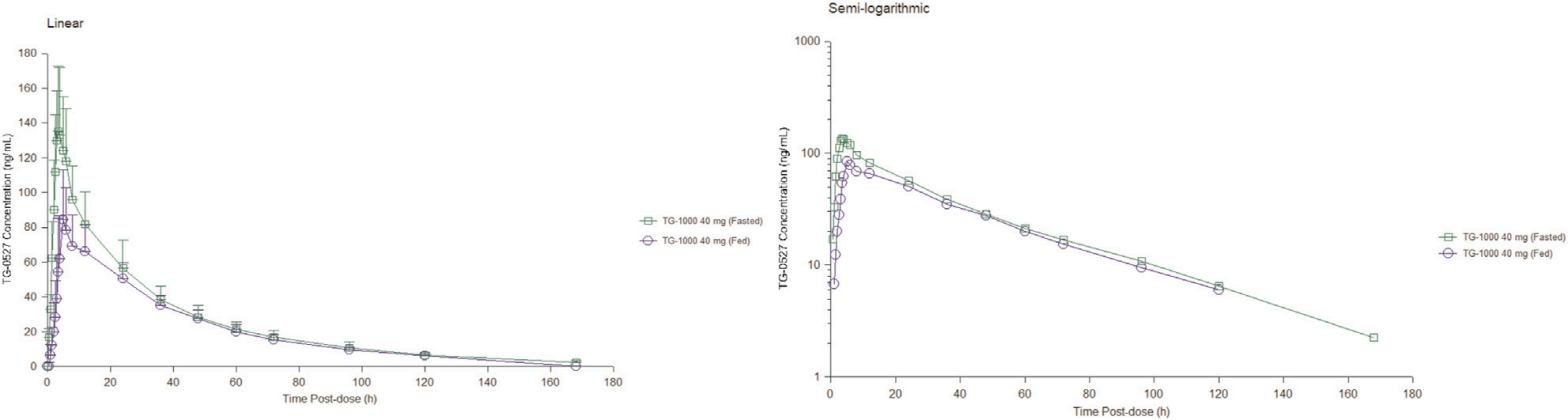
The mean plasma TG-0527 concentration-time curves under fasting and fed conditions (left: linear coordinates, right: semi-logarithmic coordinates). Mean (SD) of plasma TG-0527 concentration is displayed in this figure.

Part B; Food Effect (FE) study: After 40 mg TG-1000 capsules were administered, the PK parameters AUC_0-inf_, AUC_0-last_, and C_max_ of TG-0527 decreased by approximately 17.52%, 18.76%, and 41.35%, respectively. Additionally, compared to the fasted state, the T_max_ was delayed by approximately 1.50 h in the satiated state with a high-fat/high-calorie meal. Table 4 shows the key PK parameters of TG-0527 after a single 40 mg TG-1000 oral dose under different diet conditions, and Figure 2 displays the corresponding mean plasma drug concentration-time curves.

**TABLE 4:** The PK parameters of TG-0527 under fasting and fed conditions after administration of a single oral dose of 40 mg TG-1000.

PK parameters	Fasting (N = 12)	High-fat meal (N = 12)
AUC_0-t_ (h∙ng/mL)	4,230 (21.5)	3,490 (18.7)
AUC_0-∞_ (h∙ng/mL)	4,050 (22.6)	3,290 (20.5)
C_max_ (ng/mL)	143 (21.8)	84.1 (33.4)
T_max_ (h)^a^	3.50 (3.00, 5.00)	5.00 (3.50, 11.9)
T_1/2, z_ (h)	36.1 (7.8)	37.3 (12.1)
C_24_ (ng/mL)	54.8 (26.5)	49.6 (19.0)
CL/F (L/h)	9.46 (21.5)	11.5 (18.7)
V_z_/F (L)	493 (22.9)	617 (21.2)
CL_R_ (mL/h)	19.4 (30.3)	21.6 (27.8)
FE-C_max_ (%)		58.65 (50.65, 67.92)^a^
FE-AUC_0-t_ (%)		81.24 (72.87, 90.56)^a^
FE-AUC_0-∞_ (%)		82.48 (74.72, 91.04)^a^

AUC_0-t_ area under the concentration–time curve from time zero to the time of the last measurable concentration, AUC_0-∞_ area under the concentration–time curve from time zero to infinity, C_max_ maximum observed plasma concentration, T_max_ time to maximum plasma concentration, t_1/2_ terminal elimination half-life, V_z_/F apparent distribution volume, CL/F clearance rate, Ae cumulative excretion, Feu fractional excretion. Note: Data are expressed as geometric mean (coefficient of variation, CV%), except for T_max_, which is shown as median (min, max).

^a^ Geometric mean ratios (90% CIs) of C_max_, AUC_0-t_ and AUC_0-∞_ between high-fat meal and fasting.

### 3.3 Safety

There were no Serious Adverse Events (SAEs), Adverse Events (AEs) leading to death, or AEs resulting in permanent discontinuation from this study. Additionally, there was no significant correlation between the type, incidence, and severity of adverse events in each dose group and the dose of the study drug. Finally, there was no significant correlation between the effect of food and the type, incidence, and severity of adverse events. The safety assessment was performed based on the SP population. Table 5 summarizes the AEs that occurred in participants during the trial.

**TABLE 5:** Summary of treatment-phase adverse events (TEAE) for the study period.

	Single ascending-dose study							Food effect study	
	Placebo (N = 10)	10 mg (N = 4)	20 mg (N = 8)	40 mg (N = 8)	80 mg (N = 8)	120 mg (N = 8)	160 mg (N = 8)	40 mg fasting (N = 12)	40 mg satiating (N = 12)
TG-1000/Placebo related AEs	5 (50.0%)	2 (50.0%)	2 (25.0%)	4 (50.0%)	4 (50.0%)	4 (50.0%)	2 (25.0%)	3 (25.0%)	4 (33.3%)
Urine leukocyte positive	0	1 (25.0%)	1 (25.0%)	0	2 (25.0%)	2 (25.0%)	0	0	1 (8.3%)
Hyperuricemia	2 (20.0%)	1 (25.0%)	0	0	0	0	0	/	/
Increased total bile acid	1 (10.0%)	0	1 (12.5%)	0	0	0	0	0	1 (8.3%)
C-reactive protein is elevated	0	0	0	0	0	1 (12.5%)	0	1 (8.3%)	1 (8.3%)
Abnormal ST-T segment of ECG	0	0	0	0	1 (12.5%)	0	0	1 (8.3%)	1 (8.3%)
Elevated blood bilirubin	0	0	0	0	0	0	1 (12.5%)	/	/
Creatine phosphokinase elevation	0	0	0	1 (12.5%)	0	0	0	/	/
Increased creatinine	0	0	0	0	0	0	1 (12.5%)	/	/
Elevated white blood cell count	1 (10.0%)	0	0	0	0	0	0	/	/
Abnormal liver function	0	0	0	1 (12.5%)	1 (12.5%)	0	0	/	/
Abdominal distension	0	0	0	1 (12.5%)	0	0	0	/	/
Epigastric pain	0	0	0	0	1 (12.5%)	0	0	/	/
Hypertriglyceridemia	1 (10.0%)	0	0	0	0	0	1 (12.5%)	2 (16.7%)	2 (16.7%)
Hyperuricemia	1 (10.0%)	0	0	0	0	0	0	/	/
Anemia	0	0	0	0	1 (12.5%)	0	0	/	/

Note: *n* (%), number (incidence) of participants with an adverse.

Part A: In this part of the study, 54 participants were included in the Safety Population (SP). In the trial group, 44 participants were enrolled in the SP, with 18 participants (40.9%) experiencing 23 Treatment-phase Adverse Events (TEAEs) and 14 participants (31.8%) experiencing 18 study-drug-related TEAEs. On the other hand, 10 participants in the placebo group were enrolled in the SP, with 5 participants (50.0%) experiencing 7 TEAEs, and these 5 participants (50.0%) also experiencing 6 study-drug-related TEAEs. Among the AEs that were potentially related to the study drug (placebo or TG-1000) and occurred in more than one case in the TG-1000 trial group, “urine leukocyte positivity” in all tests was the most frequent followed by “abnormal liver function” in hepatobiliary disorders, while in the placebo group, “abnormal liver function” in all tests was the most frequent. The most frequent adverse event in the placebo group was “elevated blood uric acid” in all assays.

Part B: This part of the study had 12 participants. In the fasting phase of the study, 12 participants were enrolled, and 5 TEAEs occurred in 4 participants (33.3%), of which 4 TEAEs in 3 participants (25.0%) were study-drug related. On the other hand, 12 participants were enrolled in the satiety phase of the study, and 8 TEAEs occurred in 5 participants (41.7%), of which 7 TEAEs in 4 participants (33.3%) were study-drug related. Among the AEs potentially associated with the study drug TG-1000 and occurring in more than one case, “hypertriglyceridemia” in the metabolic and nutritional illness category during fasting and satiety phases was the most frequent.

## 4 Discussion

Although vaccination against seasonal influenza has become the primary protection method, developing vaccines for pandemic novel influenza strains takes about 6 months. In addition to its catastrophic consequences for human lives, delays in developing and distributing a global pandemic influenza vaccine could lead to social upheaval. The Neuraminidase Inhibitors (NAIs) oseltamivir and zanamivir are the current standard influenza therapy. Clinical impact has been reported to be greatest when treatment is administered early, particularly within 48 h of influenza onset. Several studies have questioned and debated the efficacy of oseltamivir in influenza treatment. Consequently, after reviewing evidence of its effect, the WHO recently decided to downgrade oseltamivir from its list of core influenza medications (Kmietowicz, 2017). Aside from the limited treatment window and effectiveness, NAIs have also been associated with the drug resistance problem (Ison, 2017). During the 2008-2009 influenza pandemic, oseltamivir-resistant H1N1 virus strains increased by more than 90% worldwide, indicating an urgent need for additional anti-flu treatments. To provide novel therapy alternatives for influenza infections, a “grab cap” mechanism has been increasingly employed in developing drugs for influenza treatment.

Baloxavixolate is the first Cap-dependent Endonuclease (CEN) small molecule drug approved for the treatment of influenza A and B infections in Japan and the U.S. Viral variations such as the I38T mutation were isolated from a small number of participants during late-stage clinical trials of baloxavixate. Although they are 20–50 times less sensitive to baloxavixolate, these mutations are not completely resistant to the drug (Ng, 2019). However, subsequent research revealed higher rates of I38X resistance, especially in pediatric participants, raising concerns regarding using baloxavixolate in treating influenza. Consequently, additional avenues for creating new CEN inhibitors with better efficacy, safety, and higher resistance barriers are required.

Based on the stable and metabolic features of TG-1000 hepatocytes *in vitro*, dogs are considered the species most related to humans. As a result, the initial dose of the phase I clinical trial was determined based on the 28-day canine toxicological experiment NOAEL. The NOAEL of TG-1000 for dogs was 60 mg/kg/day, and the equivalent dose for humans was determined to be 32.46 mg/kg, using a 0.541 equivalent dose coefficient for dogs to humans, making the total dose for a 60 kg body weight to be 1948 mg. The maximum initial dose for healthy adult volunteers was determined using the Guidelines for Estimating the Maximum Recommended Starting Dose for the First Clinical Trial of Healthy Adult Volunteers published by the Center for Drug Evaluation in 2012. The standard safety factor is usually set at ≥10, and the starting TG-1000 dose should be lower than 195 mg for every subject. However, since this is the first trial of TG-1000 on human participants, based on the preclinical evidence of extended blood drug half-life in dogs, wide tissue distribution in rats, and low total drug recovery in rat urine feces, the safety factor was increased to 200, and the proposed initial dose of TG-1000 was set at 10 mg per subject. This starting dose will ensure the safety of the participants.

This is the first human trial of TG-1000. In the Single Ascending-Dose (SAD) study, AUC was less than the proportional increase in dose, and C_max_ increased in an approximately dose-proportional manner in the 20–160 mg dose range. In the 10–160 mg dose range, T_1/2_, λz, and T_max_ of TG-0527 are dose-independent. In the 10–160 mg dose range, C_max_ and AUC implied that TG-0527 exposure increased with higher dose levels; T_1/2_ suggested that plasma TG-0527 elimination was consistent with a two-compartment model; and CL/F and CL_R_ suggested that renal clearance is not the main TG-1000 elimination route and that hepatic metabolism may be the primary clearance route (hepatic extraction rate <0.3). In the Food Effect (FE) study, T_max_ in both fasted and satiated conditions were consistent with those in the 40 mg dose group in Part A, suggesting a moderate absorption of TG-0527 *in vivo*; T_1/2_ suggested that the plasma TG-0527 elimination was consistent with the two-compartment model; CL/F and CL_R_ observed in Part B were consistent with those in Part A, suggesting that hepatic metabolism may be the primary TG-0527 elimination pathway (hepatic extraction rate <0.3). Following a high-fat diet, AUC_0-inf_, AUC_0-last_, and C_max_ of TG-0527 decreased by approximately 17.52%, 18.76%, and 41.35%, respectively. It is speculated that different meal spectra may have different effects on gastric physiology (such as gastric emptying time, gastric pH, etc.), and then affect the degree of drug absorption.

All TEAEs that occurred during this trial were classified as Grade 1-2 in severity, and all AEs were eventually resolved completely. In part A of the trial, there was no significant correlation between the type, incidence, and severity of AEs in the dose groups and the dose of the study drug. Additionally, in Part B of the study, there was no significant correlation between the type, incidence, and severity of AEs and the effects of food. The overall safety results demonstrated that no evidence of undue effects or safety signals in the present study. All the AEs potentially associated with the study drug TG-1000 were Grade 1, except for one “anemia” event of CTCAE Grade 2 in severity in the 80 mg TG-1000 dose group, which the subject tolerated and was eventually resolved completely. Positive urine leukocytes, abnormal liver function, elevated C-reactive protein, hypertriglyceridemia, and increased total bile acids are the main adverse events that occurred more than once and were potentially related to the study drug. The relevance of these adverse events to the study drug will be investigated further in future clinical trials with larger sample sizes.

## 5 Conclusion

The PK parameters have certain nonlinearity because the absorption is saturated and high-fat foods reduce its absorption. TG-1000 exhibited acceptable safety and tolerability in healthy participants. For treating influenza, it is recommended that TG-1000 should be administered under a fasting condition, but more clinical trials are needed to confirm this conclusion.

## Data Availability

The original contributions presented in the study are included in the article/Supplementary material, further inquiries can be directed to the corresponding authors.
